# Increasing Rates of Fluoroquinolone Resistance in *Escherichia Coli* Isolated From the Blood and Urine of Patients with Hematologic Malignancies and Stem Cell Transplant Recipients

**DOI:** 10.20411/pai.v1i2.115

**Published:** 2016-09-20

**Authors:** Christopher G. Hauck, Pearlie P. Chong, Melissa B. Miller, Katarzyna Jamieson, Jason P. Fine, Matthew C. Foster, Thomas C. Shea, David van Duin

**Affiliations:** 1 Division of Infectious Diseases, University of North Carolina, Chapel Hill, North Carolina; 2 Department of Pathology and Laboratory Medicine, University of North Carolina School of Medicine, Chapel Hill, North Carolina; 3 Division of Hematology/Oncology, University of North Carolina, Chapel Hill, North Carolina; 4 Biostatistics Department, Gillings School of Global Public Health, University of North Carolina, Chapel Hill, North Carolina

**Keywords:** Fluoroquinolone resistance, *Escherichia coli*, stem cell transplant, hematologic malignancy

## Abstract

Fluoroquinolone (FQ) antibiotics have been shown to reduce mortality and the number of febrile episodes when used as prophylaxis during neutropenia. Prior studies suggest that prophylaxis may result in increasing rates of FQ resistance. Fluoroquinolone non-susceptibility trends in *Escherichia coli* isolated from blood and urine cultures were evaluated over a 16-year period during which prophylaxis was initiated in patients with hematologic malignancies and stem cell transplants. Non-susceptibility rates increased after the introduction of prophylaxis, with yearly non-susceptibility rates rising from 30%–33% to 40%–88% in blood isolates. The high rates of non-susceptibility now observed raise concerns about the continued efficacy of FQ prophylaxis. This concern exists particularly in those patients undergoing stem cell transplants where the total FQ non-susceptibility rates over the study period were 82.3%. Further evaluation of the effect of FQ prophylaxis on antibiotic resistance and its efficacy in the setting of increased rates of resistance is warranted.

## INTRODUCTION

Neutropenia and the associated risk of infection is a consistent problem in the treatment of leukemia and lymphoma. Studies have shown that 12%-43% of patients undergoing treatment for leukemia have an episode of bacteremia during the course of their care [1–7]. Guidelines for the management of neutropenia in both hematologic malignancy (HM) patients and hematopoietic stem cell transplant (HSCT) recipients recommend fluoroquinolone (FQ) prophylaxis based on studies showing a significant reduction in the number of documented infections and episodes of bacteremia [7–10]. Importantly, the Infectious Disease Society of America (IDSA) neutropenia in cancer guidelines also recommend having a strategy to monitor rates of FQ resistance among gram-negative bacilli.

In this retrospective study, the primary goal was to evaluate the trend of FQ non-susceptibility rates in *E. coli* isolates from patients' blood and urine cultures at our institution from 2000 to 2015. FQ non-susceptibility rates in isolates obtained from an initial blood or urine culture from patients with HM/HSCT were compared to non-HM/HSCT patients.

## METHODS

Between 2005 and 2015, HSCT recipients and high risk patients with HM who were neutropenic (absolute neutrophil count (ANC) < 500 cells/mm^3^) received universal FQ prophylaxis (levofloxacin 500mg PO daily) per institutional protocol. Prior to 2005, use of FQ prophylaxis was at the discretion of the treating physician. Patients with HM are considered high risk if anticipated duration of neutropenia > 7 days and/or anticipated ANC < 100 cells/mm^3^. FQ prophylaxis was discontinued when the ANC was > 500 cells/mm^3^.

All *E. coli* isolates were tested for susceptibility to ciprofloxacin, levofloxacin, or both. Isolates were defined as non-susceptible if testing determined them to be either intermediate or resistant to fluoroquinolones using standardized testing and interpretive criteria as published by the Clinical and Laboratory Standards Institute. Susceptibility testing from 2000 to mid-2009 was performed using Kirby-Bauer disk diffusion. After June of 2009 susceptibility testing was performed using VITEK2 (bioMerieux, Durham, NC).

The percentage of cultures showing FQ non-susceptibility per year was calculated. Each patient was included in the evaluation only once based on their first culture positive for *E. coli* (blood or urine). Comparisons between HM/HSCT and non-HM/HSCT patients were performed with Fisher's exact or Pearson's chi-square test.

For the blood culture results, an interrupted time series analysis was performed with the objective of assessing differences in the trend in rates of FQ resistance before and after instituting a protocol of antibiotic prophylaxis for neutropenia. The HM/HSCT and non-HM/HSCT cohorts were analyzed separately using the linear regression function ‘lm’ in the software package R, which assumes independent errors. The interrupted time series model was performed using time and preprotocolized vs postprotocolized antibiotic prophylaxis as the covariates. No significant time effects were seen in the analyses, possibly due to small sample size.

## RESULTS

A total of 1324 non-HM/HSCT patients and 149 HM/HSCT (HM n = 98, HSCT n = 51) patients with blood cultures positive for *E. coli* were identified from 2000 to 2015. From 2000 to 2013 a total of 23 382 non-HM/HSCT patients and 242 HM/HSCT (HM n = 210, HSCT n = 57) patients were identified with *E. coli* positive urine cultures. The underlying malignancy diagnoses in the HM/HSCT patients are described in Table 1.

**Table 1.** Primary hematologic malignancy diagnosis of patients

Underlying Malignancy	All patients n = 355	HM Blood n = 98	SCT Blood n = 51	HM Urine n = 187	SCT Urine n = 54
Acute leukemia	116 (32)	46 (47)	23 (49)	45 (24)	16 (30)
Acute Lymphocytic Leukemia	40	12	5	23	3
Acute Myelogenous Leukemia	76	34	18	23	13
Chronic Lymphocytic Leukemia	31 (9)	7 (7)	-	26 (14)	-
Chronic Myelogenous Leukemia	16 (5)	3 (3)	2 (3)	8 (4)	4 (7)
Hodgkin Lymphoma	13 (4)	2 (2)	1 (3)	9 (5)	2 (4)
Non-Hodgkin Lymphoma	126 (35)	33 (34)	9 (17)	84 (45)	12 (22)
Large B-cell	46	13	1	34	4
Follicular	33	6	4	20	5
Marginal	10	1	-	10	-
Anaplastic	7	-	-	6	1
Burkett	9	6	1	3	-
Other	21	7	3	11	2
Myelodysplastic disorders	30 (8)	7 (7)	8 (14)	15 (8)	3 (6)
Multiple Myeloma	23 (6)	-	8 (14)	-	17 (32)

Data presented as n (%) with percent representing proportion within the column it occurs

Abbreviations: HM, hematologic malignancy; SCT, stem cell transplantation

The FQ non-susceptible rates in *E. coli* isolates in blood cultures were 81 out of 149 (54%) in the HM/HSCT group and 462/1445 (32%) in the non-HM/HSCT group (*P* < 0.001). Within the HM/ HSCT group, HSCT patients were more likely to have bacteremia caused by *E. coli* non-susceptible to FQ, compared to HM patients; 42 of 51 (82.3%) vs 39 of 98 (39.8%), *P* < 0.001.

In urine isolates the FQ non-susceptibility rates for *E. coli* isolates were 76 of 267, (28%) in the HM/HSCT group and 3408 of 23 382 (14.6%) in the non-HM/HSCT group (*P* < 0.001). Similar to the results from *E. coli* blood isolates, *E. coli* from HSCT patients displayed a higher rate of resistance (27 of 57, 47%), as compared to HM patients (49 of 210, 23%, *P* < 0.001).

**Figure 1.**
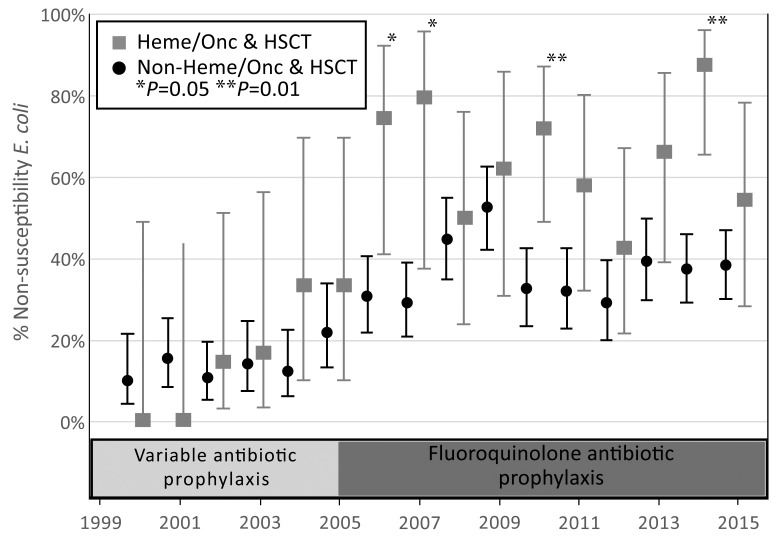
Trend in fluoroquinolone non-susceptibility rates in first *E. coli* positive blood culture by year of occurrence, 2000-2015. The error bars represent 95% confidence intervals. HSCT, hematopoietic stem cell transplant. Abbreviations: HSCT, hematopoietic stem cell transplant.

**Figure 2.**
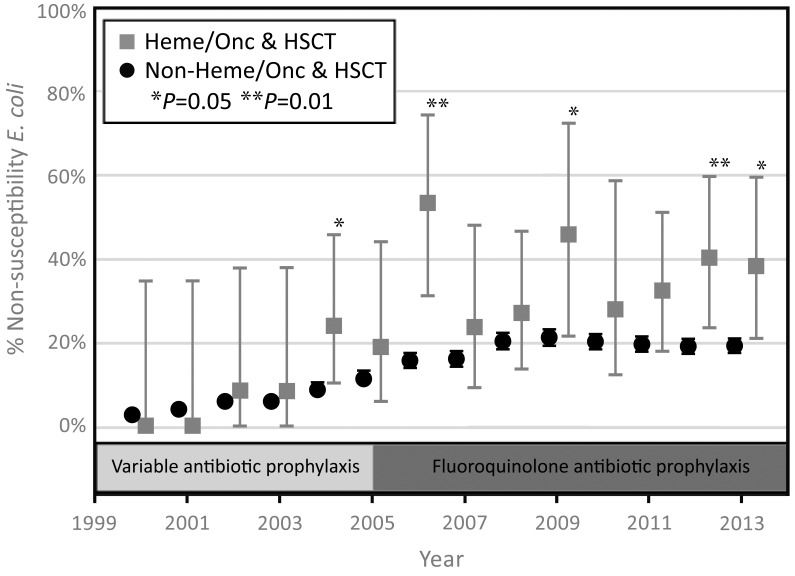
Trend in fluoroquinolone non-susceptibility rates in the first *E. coli* positive urine culture by year of occurrence, 2000-2013. The error bars represent 95% confidence intervals. Abbreviations: HSCT, hematopoietic stem cell transplant.

The annual rate of FQ non-susceptibility in *E. coli* increased across the study period of 2000-2015 for blood isolates, and the annual rate of FQ non-susceptibility in *E. coli* increased across the study for both blood and urine isolates (Figures 1 and 2). Starting in 2004 the numbers of *E. coli* that were FQ non-susceptible began to increase at a greater rate in the HM/HSCT group compared to the control group in both blood and urine culture groups. The total rate of non-susceptible *E. coli* isolates from the first positive blood cultures in the HM/HSCT group after the initiation of protocolized use of fluoroquinolone prophylaxis (2005-2015) was 63.6%, compared to 38.0% in the control group (*P* < 0.001). Over the period of 2000 to 2013 the rate of non-susceptible *E. coli* isolates from the first positive urine cultures was 33.5% in HM/HSCT in contrast to 18.3% in the control group (*P* < 0.001). Additionally, the absolute number of FQ non-susceptible *E. coli* isolates from the first blood cultures in the HM/HSCT group increased from an average of 0.8 cases/year prior to 2005, to 7.0 cases/year from 2005-2015. The average number of FQ non-susceptible *E. coli* isolates from the first urine cultures showed a similar trend with 1.7 cases/year from 2000-2004 and 6.7 cases/year from 2005-2013.

An interrupted time series analysis was used to compare trends in FQ resistance before and after the initiation of protocolized antibiotic prophylaxis in 2005. Linear regression analyses were performed with the inclusion of the covariate of time. Comparison of the trends in non-susceptibility rates prior to 2005 for HM/HSCT and non-HM/HSCT were not statistically significant (*P* = 0.23). A comparison of the trend between the two groups after 2005 was just outside the margin of statistical significance (*P* = 0.06). Within groups, a comparison of before vs after 2005 was significant in the HM/HSCT group (*P* = 0.003) and not significant for the non-HM/HSCT group (*P* = 0.10), indicating that the only significant change in the trend of antibiotic resistance rates occurred in the HM/HSCT group after initiation of antimicrobial prophylaxis.

## DISCUSSION

This trend of increasing FQ non-susceptibility in the HM/HSCT group presents questions about the efficacy of FQ antibiotics for neutropenic prophylaxis. The marked increase in *E. coli* non-susceptible to FQ from 2005 to 2007 in the HM/HSCT group is of particular concern because prophylaxis with levofloxacin became part of the standard neutropenia protocol in 2005. In prior studies looking at rates of FQ non-susceptibility after the introduction of prophylaxis, similar increases in non-susceptibility were seen in some [11–14], while others found no change in rates of non-susceptibility after the onset of prophylaxis [15–17]. The rise in resistance seen in this study is most likely an effect of increased FQ exposure, with the more significant rise seen in the HM/HSCT group likely reflecting the initiation of FQ prophylaxis. The observed highest rates of resistance in HSCT patients probably reflects increased FQ exposure, as these patients generally have had multiple neutropenic episodes.

Another concern pertains to the efficacy of prophylaxis in the setting of high baseline rates of FQ resistance. It is unknown whether the evidence for the benefit of prophylaxis continues to hold true in the setting where non-susceptibility rates are 40%-60%. This uncertainty exists particularly in the HSCT population where 82.3% of *E. coli* isolates were non-susceptible to FQ. However, these findings may reflect successful suppression of FQ-susceptible isolates, such that only FQ non-susceptible organisms cause breakthrough infections.

In conclusion, FQ non-susceptibility rates in *E. coli* isolated from blood and urine cultures increased over the study period, especially in patients with hematologic malignancies and HSCT patients. This increase in rates of FQ non-susceptibility coincided with the implementation of levofloxacin prophylaxis for patients with neutropenia at our institution. Whether this is a reversible trend remains to be determined.
